# The hysterectomy: influence of the surgical method in benign disease on convalescence and quality of life

**DOI:** 10.1007/s00404-022-06778-9

**Published:** 2022-10-27

**Authors:** Saskia Spaich, Christel Weiss, Sebastian Berlit, Amadeus Hornemann, Marc Sütterlin

**Affiliations:** 1 Department of Obstetrics and Gynaecology, University Medical Centre Mannheim, Heidelberg University, Theodor-Kutzer-Ufer 1-3, 68167 Mannheim, Germany; 2 Department of Medical Statistics and Biomathematics, University Medical Centre Mannheim, Heidelberg University, Mannheim, Germany; 3 Department of Obstetrics and Gynaecology, Krankenhaus Sachsenhausen, Frankfurt, Germany

**Keywords:** (Vaginal) hysterectomy, TLH, LASH, Quality of life, Postoperative course

## Abstract

**Purpose:**

The aim of this study was to evaluate the postoperative course after different methods of hysterectomy for benign diseases with special emphasis on time to recovery and patient-centred aspects such as postoperative quality of life and satisfaction.

**Methods:**

A collective of 242 women who had undergone vaginal hysterectomy (VH), laparoscopic supracervical hysterectomy (LASH) or total laparoscopic hysterectomy (TLH) for various benign conditions was studied in this retrospective investigation. Patients completed a standardised questionnaire addressing quality of life, recovery and sick leave as well as general questions on their postoperative course after hysterectomy.

**Results:**

A total of 242 cases were analysed (82 VH, 92 LASH and 68 TLH). The data demonstrate significant differences in regard to age between groups. The present study shows shorter hospitalisation with laparoscopy, with LASH patients returning to work at least one week earlier on average. There were no relevant differences in the overall postoperative course during the index hospital stay. In the long run, laparoscopic patients were not more satisfied with their choice than VH patients.

**Conclusion:**

No significant long-term differences could be observed in terms of quality of life and overall postoperative satisfaction between VH and LH groups. In regard to socioeconomic aspects, laparoscopic approaches were associated with shorter hospitalisation and LASH patients returning to work at least one week earlier on average. Contrary to these data on objective recovery; however, a laparoscopic approach did not lead to patient-perceived, i.e. subjective improvement of time to full recovery.

## What does this study add to the clinical work

**Table:** 

No significant long-term differences could be observed in terms of quality of life, sexuality and overall postoperative satisfaction when different methods of hysterectomy were compared. In regard to socioeconomic aspects, laparoscopic approaches were associated with shorter hospitalisation and LASH patients returning to work earlier.	

## Introduction

Hysterectomy (HE) is one of the most frequent surgical intervention in operative gynecology. The lifetime probability of having a hysterectomy as a woman in Germany is around 17% [1]. In 2005, around 153,000 women in Germany received a hysterectomy [2]. The same analysis demonstrated a nationwide hysterectomy rate of 3.6 per 1,000 person-years with pronounced regional differences. Therefore, it is essential to continuously optimise the surgical strategies with special emphasis on patient-related outcome.

Vaginal hysterectomy (VH) and laparoscopic hysterectomy (LH) confer significant advantages over abdominal hysterectomy as they have been demonstrated to benefit patients in regard to fewer perioperative complications, shorter length of hospital stay, improved short-term quality of life (QoL) and faster return to normal activities [3]. With few exceptions VH and LH have become the methods of choice.

The indication for a hysterectomy can derive from various reasons. In a first step, the decision is based on benign versus malignant disease. Benign causes make up about 90% of cases [1]. According to an evaluation in 2012, fibroids were the most common indication for surgery in benign disease with almost 61% of cases. These were followed by a large margin by subsidence complaints with 28%, bleeding disorders with 25% and endometriosis with 15% (10). Concerning LH for benign reasons, both subtotal as well as total extirpation of the uterus have evolved to standard surgical procedures.

Thus, the aim of this study was to evaluate the postoperative course after different methods of hysterectomy in benign disease, with special emphasis on time to recovery as well as patient-centred outcomes such as subsequent quality of life and satisfaction.

For this purpose, a collective of women who had undergone vaginal hysterectomy (VH), laparoscopic supracervical hysterectomy (LASH), or total laparoscopic hysterectomy (TLH) for various benign conditions were studied.

## Methods

### Patient population and methodology

All patients who had been subjected to HE (LASH, TLH, VH) in the Department of Obstetrics and Gynaecology of the University Medical Centre Mannheim, Germany, between 2010 and 2015 were retrospectively identified in the database. As a result, 468 patients were identified and contacted once 2016. A questionnaire was enclosed for data collection. Complementing the questionnaire data set, additional data derived from the original inpatient stay were acquired. All patients provided written informed consent. Ethical approval was obtained through the Ethics Committee II of Heidelberg University, Medical Faculty Mannheim (2015-629 N-MA).

#### Contents of questionnaire

##### General questions

The aim of the first part was to obtain, complement and validate general characteristics.

##### Well-being since hysterectomy

These questions asked about the current state of health and its influence on professional and private life. In addition, patients were asked about the development of well-being after surgery. These questions were adapted from the Short-Form Health Survey (SF-12) questionnaire of the World Health Organization (WHO).

##### Recovery and sick leave

Patients were asked about when they had returned to their jobs or activities, what influence pain after surgery had had on their return to work, and after what time they had felt that they had fully recovered.

##### Postoperative course

The data collection on the postoperative course included questions about pain, vaginal bleeding, menopausal symptoms, and weight changes.

##### Sexuality

Questions were asked about sexual dysfunction before surgery, changes in sexual dysfunction after surgery, and newly perceived dysfunction.

Finally, overall satisfaction with the surgical outcome was analysed.

### Statistics

An Excel spreadsheet was created to document the responses obtained, which was imported into the statistical program SPSS. An analysis of variance (ANOVA) was performed for continuous target variables, the Kruskal–Wallis test for discrete and ordinal-categorical target variables, and the chi-square test for categorical target variables. In general, a test result with a p-value less than 0.05 was considered statistically significant.

If the null hypothesis (no influence of the operation method on the target variable) was rejected, post hoc tests were undertaken between two procedures in each case. If equality of variance could be assumed, a post hoc analysis according to Hochberg was performed. If this was not the case, a post hoc analysis according to Games-Howell was performed.

## Results

A total of 468 women who had undergone hysterectomy due to benign disease at the University Hospital Mannheim, Germany, were identified and asked to complete the attached questionnaire. Fifty-two patients (11%) could not be reached by mail at all. In total, we received 248 responses without significant differences between modes of surgery. Six questionnaires could not be evaluated because of incomplete answers, resulting in 242 (82 VH, 92 LASH, 68 TLH) complete questionnaires for further analyses.

Detailed patient characteristics including age, BMI, parity, duration of surgery, uterus weight, length of inpatient hospital stay and previous operations are presented in Table 1.

**Table 1:** Demographic parameters

Variable	All *n* = 242 Median (min–max)	VH *n* = 82 Median (min–max)	LASH *n* = 92 Median (min–max)	TLH *n* = 68 Median (min–max)	Global analysis* p-value	pairwise analysis** p-value
Age (years)	48 (33–84)	54 (33–84)	46 (33–81)	48 (33–74)	< 0.001	VH vs. LASH: < 0.001 VH vs. TLH: < 0.001
BMI (kg/m^2^)	25.2 (18.4–47.6)	25.4 (18.4–46)	24.6 (18.5–44.9)	26.1 (19.5–47.6)	n.s	
Uterus weight (g)	117 (21–1320)	87 (25–247)	180 (27–1320)	144 (21–684)	< 0.001	VH vs. LASH: < 0.001 VH vs. TLH: < 0.001 LASH vs. TLH: < 0.05
Inpatient length of stay (d)	2 (1–28)	4 (1–28)	2 (1–7)	2 (2–6)	< 0.001	VH vs. LASH: < 0.001 VH vs. TLH: < 0.001
Parity	2 (0–8)	2 (0–8)	2 (0–6)	2 (0–3)	< 0.001	VH vs. LASH: < 0.001 VH vs. TLH: < 0.001
Previous abdominal surgery	1 (0–6)	1 (0–4)	1 (0–6)	1 (0–5)	n.s	

*VH* vaginal hysterectomy, *LASH* laparoscopic supracervical hysterectomy, *TLH* total laparoscopic hysterectomy, *BMI* body mass index

*p values based on ANOVA, Kruskal–Wallis test and *χ*^2^ test

**If the global analysis was significant, pairwise comparisons were made and corresponding *p* values reported

Patients’ median age at time of surgery was 48 years with individual surgical methods differing significantly from each other in terms of age (*p* < 0.001). The VH group, with a median age of 54 years, was significantly older than patients in both laparoscopic groups (*p* < 0.001), both of which did not differ from each other (*p* = 0.304). The average BMI of the patients at time of surgery was 26.5 kg/m^2^ with no significant differences between groups (*p* = 0.054).

The majority of patients (88.9%) had received vocational training. All studied groups had in common that non-academic training was the most frequent form of vocational training reported. No statistically significant correlation could be established for the observed differences (*p* = 0.19).

The median uterine weight was 117 g. There were significant differences between the groups (*p* < 0.001). The post hoc tests confirmed significant differences between all procedures, with highly significant disparities between VH and the laparoscopic procedures (*p* < 0.001). Pertaining to the observed median, uteri weighed the least in VH (87 g), followed by TLH (144 g) and LASH (180 g). Congruently, the heaviest uteri were recorded in the laparoscopic groups. In the LASH group, two uteri weighed even more than 1 kg.

## Previous medical history and comorbidities

While the median number of previous deliveries was two, significant differences were found between the groups (*p* < 0.001). VH patients had significantly more births than laparoscopic patients (*p* < 0.001), where both subgroups did not differ from each other (*p* = 0.99). The distribution of parities also showed that the proportion of childless patients was highest in the group with laparoscopic procedures (LASH 25%, TLH 20.6%, VH 4.9%).

In our patient population, the number of previous abdominal surgeries did not differ significantly from each other across groups (*p* = 0.058).

There were significant differences between the groups in terms of arterial hypertension (*p* < 0.01). With 45% in the VH group, arterial hypertension was significantly more frequent than in LASH/TLH patients (*p*1 < 0.01, *p*2 < 0.03). There were no significant differences between LASH and TLH (*p* = 0.98).

For all other chronic diseases such as diabetes mellitus, dyslipidemia, thyroid disease, heart disease, bronchial asthma, lung disease and bleeding or thrombotic events, there were no significant differences between the groups.

### Symptoms before surgery/hysterectomy

Problems with prolapse had occurred in about 21% of all patients before the operation. Significant differences were observed between the groups (*p* < 0.001). VH patients were operated on significantly more often (52%) due to symptoms of prolapse (*p* < 0.001), followed by LASH (8%) and TLH (1%)—(*p* < 0.05 for the latter comparison).

Abdominal pain had affected almost a quarter of all patients (25%). There were no significant differences between the groups (*p* = 0.12).

Bleeding disorders were reported as the most frequent symptom before HE. Significant differences were found between the groups (*p* < 0.001). Preoperative bleeding disorders were significantly more frequent in the TLH and the LASH group than in the VH group (*p* < 0.001), while there was no significant difference between TLH and LASH (*p* = *p* = 0.48)—see Table 2.

**Table 2:** Symptoms before surgery/hysterectomy

Variable	All *n* = 242	VH *n* = 82	LASH *n* = 92	TLH *n* = 68	Global analysis* p-value	pairwise analysis** p-value
Descensus symptoms	Yes: 21% No: 79%	Yes: 52% No: 48%	Yes: 8% No: 92%	Yes: 1% No: 99%	< 0.001	VH vs. LASH: < 0.001 VH vs. TLH: < 0.001 LASH vs. TLH: < 0.05
Abdominal pain	Yes: 25% No: 75%	Yes: 17% No: 83%	Yes: 30% No: 70%	Yes: 26% No: 74%	n.s	
Bleeding disorders	Yes: 46% No: 54%	Yes: 29% No: 71%	Yes: 51% No: 49%	Yes: 71% No: 29%	< 0.001	VH vs. LASH: < 0.01 VH vs. TLH: < 0.001

*VH* vaginal hysterectomy, *LASH* laparoscopic supracervical hysterectomy, *TLH* total laparoscopic hysterectomy

*p values based on *χ*^2^-test

**If the global analysis was significant, pairwise comparisons were made and corresponding *p* values reported

### General health condition

The vast majority of patients was positive about their general health at the time of the interview (84%). However, there were significant differences between the groups (*p* < 0.02). LASH patients rated their health status significantly better than VH patients (*p* < 0.01), see Table 3. Following up on the question about their general state of health, the patients were asked about limitations in physical activities. At the time of survey, 69% stated that they were not limited in their daily life. Yet, significant differences were found between the groups (*p* < 0.01), VH patients felt significantly more restricted than LASH patients (*p* < 0.01)—see Table 4.

**Table 3:** Current general (subjective) state of health at the time of the interview

Healthy	All *n* = 239	VH *n* = 82	LASH *n* = 90	TLH *n* = 67
Very well	29%	24%	37%	24%
Well	55%	50%	54%	61%
Moderate	13%	20%	7%	13%
Bad	3%	5%	2%	1%
Very Bad	0%	1%	0%	0%

*VH* vaginal hysterectomy, *LASH* laparoscopic supracervical hysterectomy, *TLH* total laparoscopic hysterectomy

More than three-quarters (84%) of the patients were positive about their general health. There were significant differences between the groups *(p* <0.02,  *p* values based on Kruskal–Wallis test). LASH patients rated their health status significantly better than VH patients (Post hoc *p* < 0.01)

**Table 4:** Restriction in physical activities

Restriction	All *n* = 240	VH *n* = 81	LASH *n* = 91	TLH *n* = 68	Global analysis* p-value	pairwise analysis** p-value
None	69%	58%	80%	66%	*p* < 0.01	VH vs. LASH: < 0.01
Moderate	22%	23%	15%	29%	n.s	
Severe	9%	19%	3%	4%	n.s	
Very severe	0%	0%	1%	0%	n.s	

*VH* vaginal hysterectomy, *LASH* laparoscopic supracervical hysterectomy, *TLH* total laparoscopic hysterectomy

*p values based on Kruskal–Wallis test

**If the global analysis was significant, pairwise comparisons were made and corresponding *p* values reported

After hysterectomy, about three quarters of the patients experienced an improvement in well-being (75%—"Improved" or "Strongly improved"). Common to all groups was that “improvement” was the most frequent change (40%), followed by “strong improvement” (35%), “no change” (21%) and worsening (3%—"Worsened" and "Strongly worsened").

No significant correlation could be established for the observed differences between the groups (*p* = 0.47). Regarding the questions on the postoperative course covered by the short-form-healthy-survey no significant differences were found between groups. Details are presented in Table 5.

**Table 5:** Short-form-health-survey

	All *n* (%)	VH *n* (%)	LASH *n* (%)	TLH *n* (%)
I. Since the hysterectomy I was happy and in good spirits:				
Always	44 (19%)	17 (22%)	11 (13%)	16 (25%)
Usually	144 (63%)	6 (8%)	59 (69%)	39 (60%)
More often	25 (11%)	46 (60%)	12 (14%)	7 (11%)
Rarely	12 (5%)	5 (6%)	4 (5%)	3 (5%)
Never	3 (1%*)*	3 (4%*)*	0 (0%*)*	0 (0%)
II. Since the hysterectomy I have felt calm and relaxed:				
Always	49 (22%)	19 (25%)	14 (17%)	16 (25%)
Usually	123 (55%)	41 (55%)	51 (61%)	31 (48%)
More often	35 (16%)	7 (9%)	15 (18%)	13 (20%)
Rarely	12 (5%)	5 (7%)	3 (4%)	4 (6%)
Never	4 (2%)	3 (4%)	1 (1%)	0 (0%)
III. Since the hysterectomy I have felt active and full of energy:				
Always	36 (16%)	12 (16%)	11 (13%)	13 (20%)
Usually	118 (53%)	36 (49%)	51 (60%)	31 (48%)
More often	49 (22%)	16 (22%)	16 (19%)	17 (26%)
Rarely	16 (7%)	6 (8%)	6 (7%)	4 (6%)
Never	4 (2%)	3 (4%)	1 (1%)	0 (0%)
IV. Since the hysterectomy I have felt fresh and rested when I woke up:				
Always	34 (15%)	12 (16%)	11 (13%)	11 (17%)
Usually	104 (47%)	38 (52%)	40 (48%)	26 (41%)
More often	49 (22%)	12 (16%)	18 (22%)	19 (30%)
Rarely	29 (13%)	8 (11%)	13 (16%)	8 (13%)
Never	4 (2%)	3 (4%)	1 (1%)	0 (0%)
V. Since the hysterectomy I have been able to take an interest in the life around me:				
Always	96 (44%)	31 (42%)	33 (40%)	32 (50%)
Usually	93 (42%)	30 (41%)	40 (48%)	23 (36%)
More often	20 (9%)	7 (10%)	6 (7%)	7 (11%)
Rarely	6 (3%)	3 (4%)	2 (2%)	1 (2%)
Never	5 (2%)	2 (3%)	2 (2%)	1 (2%)

All comparisons not significant (n.s.), *p* values based on Kruskal–Wallis test

### Recovery and sick leave

The median length of hospital stay after hysterectomy was 2 days. A significant difference was found between groups (*p* < 0.001): VH patients remained hospitalised significantly longer, with a median of 4 days (*p* < 0.001), while there were no differences between LASH and TLH—both 2 days median length of hospital stay (*p* = 0.949).

The average duration until resumption of professional or private physical activities was 4.6 weeks. Significant differences were found between groups (*p* < 0.01). LASH patients resumed their activities significantly earlier than VH and TLH (p1 < 0.01, p2 < 0.05), on average 1 week earlier (Table 6). The small number of patients (*n* = 6) with a time period of more than 12 weeks who did not resume their activities were excluded from the calculation of the average. These six patients had chosen this answer because they had already been retired. The average subjective recovery time after surgery was 6.2 weeks. No statistical correlation could be established for the observed differences between the groups (*p* = 0.64). The average duration of sick leave was 4.4 weeks or 31 days without significant differences between the collectives (*p* = 0.11).

**Table 6:** Time to return to work (mean value in weeks)

Variable	All Mean ± SD	VH Mean ± SD	LASH Mean ± SD	TLH Mean ± SD	Global analysis* p-value	pairwise analysis** p-value
Time to return to work (in weeks) Mean ± SD	4.6 ± 2.4	5.2 ± 2.4	3.9 ± 2.3	5.0 ± 2.3	< 0.01	VH vs. LASH: < 0.01 VH vs. TLH: < 0.001 LASH vs. TLH: < 0.05

*VH* vaginal hysterectomy, *LASH* laparoscopic supracervical hysterectomy, *TLH* total laparoscopic hysterectomy

* p values based on Kruskal–Wallis test

**If the global analysis was significant, pairwise comparisons were made and corresponding *p* values reported

About three quarters of the patients felt that the length of their sick leave was appropriate. No statistical significance could be established for the observed differences in sick leave between the three groups (*p* = 0.09). Of the patients who felt that the length of their sick notes was not appropriate, 83% wished for a longer sick note, while 17% would have been satisfied with a shorter sick note. This patient-centred quantification of time to recovery would result in a calculated additional sick leave of 2.4 days on average in the group of patients who desired a longer duration of their sick leave.

### Postoperative course

About 72% of patients reported pain after the operation. When pain occurred, duration of 1–3 days was commonly reported in all groups. No significant difference was found between groups (*p* = 0.63). About 40% of patients reported postoperative vaginal bleeding within a time period of 8 weeks after surgery. Significant differences were found between the groups (*p* < 0.01), with bleeding occurring significantly less frequently and for a shorter time after LASH compared to VH and TLH (p1 < 0.01, p2 < 0.05)—see Table 7.

**Table 7:** Postoperative course

Variable	All *n* = 242	VH *n* = 82	LASH *n* = 92	TLH *n* = 68	Global analysis* p-value	pairwise analysis** p-value
Descensus symptoms	Yes: 8% No: 92%	Yes: 10% No: 90%	Yes: 5 No: 95%	Yes: 10% No: 90%	n.s	
Abdominal pain	Yes: 72% No: 28%	Yes: 73% No: 27%	Yes: 73% No: 27%	Yes: 67% No: 33%	n.s	
Bleeding (< 8 weeks post surgery)	Yes: 40% No: 60%	Yes: 47% No: 53%	Yes: 27% No: 73%	Yes: 50% No: 50%	< 0.01	VH vs. LASH: < 0.01 LASH vs. TLH: < 0.05

*VH* vaginal hysterectomy, *LASH* laparoscopic supracervical hysterectomy, *TLH* total laparoscopic hysterectomy

*p values based on Kruskal–Wallis test and *χ*^2^-test

**If the global analysis was significant, pairwise comparisons were made and corresponding p values reported

### Sexuality

The effect of the operation on sexual life was unanimous. More than half of the patients did not notice any effects on their sexual life, and there were no significant differences between groups (*p* = 0.48). About 55% of patients reported having been sexually active within 4 weeks prior to the operation. There were no significant differences between groups (*p* = 0.79). Among the patients who had not been sexually active before the operation, the majority stated that physical complaints had not been the cause. There were no significant differences between the groups (*p* = 0.25).

In the time period since surgery, about 84% of the patients were sexually active. There were significant differences between the groups (*p* < 0.01). TLH patients were significantly more sexually active than patients from the VH group (*p* < 0.03), while there were no significant differences between VH and LASH (*p* = 0.053). Of the patients who were sexually active in the time period since the operation, 13% reported new pain and 10.5% reported new sensory disturbances during sexual intercourse. No significant differences were observed between the groups (*p* = 0.12 or *p* = 0.65). Half of the patients reported an improvement of existing symptoms during sexual intercourse. No change was seen in 36% of patients, while 14% experienced a worsening, with no significant differences between groups (*p* = 0.70).

### Overall satisfaction

Finally, patients were asked about their overall satisfaction with the outcome of the operation. Reassuringly, 66% of the patients were "very satisfied" and 29% were "satisfied” meaning that the vast majority (95%) of patients were either very satisfied or satisfied with the result of the operation with only a very small fraction being “moderately satisfied” (3%) and an even smaller fraction reporting dissatisfaction (2%) with the surgical result. There were no significant differences between the groups (*p* = 0.06)—see Table 8.

**Table 8:** Satisfaction with the surgery

Satisfaction	All *n* = 238	VH *n* = 80	LASH *n* = 90	TLH *n* = 68
Very satisfied	156 (66%)	45 (56%)	64 (71%)	47 (69%)
Satisfied	68 (29%)	27 (34%)	22 (25%)	19 (28%)
Moderately satisfied	8 (3%)	5 (6%)	2 (2%)	2 (2%)
Dissatisfied	3 (1%)	2 (3%)	1 (1%)	0 (0%)
Very dissatisfied	3 (1%)	1 (1%)	1 (1%)	1 (1%)

*VH* vaginal hysterectomy, *LASH* laparoscopic supracervical hysterectomy, *TLH* total laparoscopic hysterectomy

*p* = 0.06 based on Kruskal–Wallis test, no significance

## Discussion

The median age in this study was 48 years and was thus similar to the results of a number of other studies [1, 4, 5]. However, there were significant differences in age between the groups with VH and laparoscopic procedures in our study (Table 1). This finding is generally congruent with data from the other trials [5] with differences between the VH group and the laparoscopic groups being a little more pronounced in our study.

One conceivable reason for the higher age of the VH group could be that the indications for surgery differed from laparoscopic approaches, with some symptoms (e.g. prolapse) appearing at later times in life or being tolerated for longer periods of time before surgery, while bleeding disorders are usually causing younger patients to consider HE.

Overall, the most frequent symptoms leading to HE were bleeding disorders, which occurred in almost every second patient in our study. Affected patients underwent laparoscopic surgery significantly more often (Table 2), offering a potential explanation for the younger patient age in the LH groups. On the other hand, descensus and prolapse symptoms are generally associated with a history of giving birth(s) and may thus partially explain the finding of higher patient age in the VH group. Furthermore, the VH approach allows for complementing surgical procedures addressing these symptoms. In line with this observation, prolapse served as indication for HE in only 20% of cases of our study, while it amounted to even 50% in the subgroup of VH patients (Table 2). In support of this notion, multiple publications show a decrease in pelvic floor function with increasing age and/or parity [6–8].

While the LASH group was operated on more often due to prolapse as compared to the TLH group, age is not an explanatory factor here. In this regard, it is conceivable that patients were advised to undergo LASH with the ulterior motive that keeping the cervix in place would reduce the likelihood of further prolapse symptoms and retain the option for future surgical interventions aimed at symptom alleviation in this regard, although the literature contains several studies that have compared total and subtotal HE through many years of follow-up, with no long-term benefits from leaving the cervix in place [9–11]. Overall, HE itself does not seem to increase the risk of developing symptoms of descent; rather, the development of descent is dependent on pre-existing limitations in pelvic floor function [12]. These data are corroborated by our findings of similar numbers of prolapse symptoms in the postoperative course across all surgical groups.

There were significant differences in uterine weights between the groups with VH and laparoscopic procedures (Table 1). Uterine weight decreases over a lifetime due to atrophy after menopause [13] partially explaining the finding, that weight of uteri was found to be lowest in the VH group which generally represented older patients in our study. Congruently, the maximum uterine weight in this study was 247 g for the VH, while it was exceeded about 5 times by specimen of laparoscopic procedures. In line with this observation, other studies also identified VH uteri as the lightest [14]. Although laparoscopic approaches might have been primarily preferred in heavy uteri (especially due to fibroids) when the surgical approach was chosen, the literature shows both VH and LH approaches to be generally feasible for heavy uteri [15–17] [18].

The fact that VH patients rated their current health status significantly worse than LASH patients cannot be fully explained by the difference in age because no significant difference regarding this status was found between VH and TLH patients, despite the younger age in the TLH group. There were no significant differences between LASH and TLH in terms of self-reported current health status. However, HE had a positive effect on patients' quality of life in the vast majority of cases, with no differences between procedures.

### Socio-economic ramifications

Major findings of this trial pertain to length of hospital stay as well as subjective and objective time to recovery.

The median length of stay of a patient undergoing HE was 2 days with significant differences between VH and TLH/LASH (Table 1). Hospitalisation after LH was 2 days shorter than for VH. A shorter length of stay after laparoscopic surgery was also found in other studies [19–21]. As in the majority of other publications, no significant differences were found between LASH and TLH.

The shortest time to return to daily activities was reported in the LASH group. Patients in this group resumed their jobs or activities an average of one week earlier. Significant differences were found between LASH and VH as well as LASH and TLH, while VH and TLH did not differ (Table 6). The reason for these objective discrepancies is unclear, as LASH patients did not feel they recovered earlier than the rest of the patients. While the fact that LASH patients were significantly younger than VH patients may have contributed to the observation that LASH patients showed the shortest time to resumption of physical activities, age does not seem to be the only explanation due to the respective similarity of the laparoscopic groups. Therefore, it is possible that the comparatively least invasive LASH was the main reason for the shortest abstinence from work. It is conceivable that leaving the cervix in place also conferred a psychological advantage, which may have caused patients to start work earlier.

The subjective (i.e. patient-perceived) recovery time was significantly longer than the 4.6 weeks of sick leave, namely 6.2 weeks. No significant differences were found between the groups with regard to the duration of sick leave. Despite the fact that this was significantly shorter than the subjective recovery time, around 75% of the patients still felt that the duration of their sick leave had been appropriate. Among the patients whose sick leave did not coincide with their individual time to full recovery, more than 80% wanted an extension. In a study by Vonk Noordegraaf et al. [22], a “considered medically possible” activity build-up was worked out depending on the surgical method. In this protocol, a classification was made into light, moderate and heavy activity; the highest level was determined to be resumption of work. Regarding the latter, a period of 3–4 weeks (LASH 3 weeks, TLH 3–4 weeks, VH 4 weeks) was considered possible, which roughly corresponds to the feedback from our patients.

In summary, laparoscopic procedures may offer benefits in terms of hospitalisation and recovery. However, the discrepancy between objective and subjective time to recovery across all groups needs to be elucidated by future studies.

### Sexuality

In this work, there were no significant differences between the groups with regard to postoperative sexuality. The majority stated that the surgery had had no effect on their sex life. Within those groups of patients that had reported sexual dysfunction prior to surgery, similar benefits were observed across all groups independent of the surgical approach. Overall, sexual activity as reported by patients increased from 55% within 4 weeks prior to surgery to 84% in the postoperative course. Nevertheless, it remains unclear, whether this observation derives from different time spans analysed or real changes in sexual activity. In principle, the effects of hysterectomy on sexuality can be considered positive [23]. Similar effects were also observed by Radosa and colleagues; here, positive effects on sexual life were shown regardless of the choice of method, which is corroborated by our data [24]. Preservation of the cervix does not appear to be an advantage, which was also observed in other studies [25–27].

### Overall satisfaction

The vast majority of patients in our patient population reported satisfaction with their decision to have surgery. Many current studies confirm a general improvement in quality of life after hysterectomy [28–31]. Gorlero and colleagues reported advantages of LASH over TLH over the first 12 months. However, the majority of studies did not show advantages of any surgical method with regard to postoperative well-being [32]. Congruently, no significant differences were found in this work. The dissected differences in subanalyses between the individual approaches do not seem to have affected the overall satisfaction with the surgery.

## Study limitations

Our study has a number of relevant limitations and caveats. It is evident that VH patients differed in their baseline characteristics from the laparoscopic patients, which has to be kept in mind when comparing the results. These differences primarily include age (with minor associated comorbidities), indication, parity and uterine weight. Further limitations arise from the retrospective character of the survey itself: the study design entails the risk of selection bias as well as recall bias. Exemplarily, more severely affected patients may not have taken part in the study. Furthermore, memories of the course of treatment are sure to have been subject to certain distortions, since the time between the operation and the survey was up to 5 years. The study’s significance is also limited by the lack of control groups. Furthermore, potential secondary interventions such as ovariectomy or colporrhaphy were not taken into account in the data collection, so that these cannot be excluded as confounding factors.

## Conclusion

While VH patients in our study were significantly older than patients treated with LH, no significant long-term differences could be observed in terms of quality of life, sexuality and overall postoperative satisfaction. In regard to socioeconomic aspects, laparoscopic approaches were associated with shorter hospitalisation and LASH patients returning to work at least one week earlier on average. Contrary to these data on objective recovery; however, a laparoscopic approach did not lead to patients feeling that they recovered more quickly.

## Abbreviations

HE: Hysterectomy
LH: Laparoscopic hysterectomy
VH: Vaginal hysterectomy
TLH: Total laparoscopic hysterectomy
LASH: Laparoscopic supracervical hysterectomy
BMI: Body mass index
Qol: Quality of Life
